# 136. Attitudes and Practices of Antimicrobial Resistance and Antimicrobial Stewardship at the Uganda Cancer Institute

**DOI:** 10.1093/ofid/ofab466.338

**Published:** 2021-12-04

**Authors:** Elizabeth Gulleen, Margaret Lubwama, Alfred Komakech, Elizabeth M Krantz, Catherine Liu, Warren Phipps

**Affiliations:** 1 Fred Hutchinson Cancer Research Center, Seattle, Washington; 2 Makerere University, Kampala, Kampala, Uganda; 3 Uganda Cancer Institute, Kampala, Kampala, Uganda; 4 Fred Hutch Cancer Research Center, Seattle, Washington; 5 Fred Hutchinson Cancer Research Center; University of Washington, Seattle, Washington

## Abstract

**Background:**

As access to cancer treatment has increased in sub-Saharan Africa (sSA), infection-related complications are a growing concern. Little is known about infection management practices in this setting. Understanding the unique challenges to diagnosing and treating infections can inform the development of targeted strategies to improve infection management for cancer treatment programs throughout sSA.

**Methods:**

We conducted a cross-sectional survey of doctors, nurses, and pharmacists at the Uganda Cancer Institute (UCI), a national cancer referral hospital in Kampala, Uganda. The 25-item survey was designed to assess staff knowledge of antimicrobial resistance and antimicrobial stewardship, investigate antibiotic decision-making practices, and identify barriers to diagnosing and treating infections.

**Results:**

Of the 61 respondents, 25 (41%) were doctors, 7 (11%) were pharmacists, and 29 (48%) were nurses. In total, 98% (60/61) had heard of the term “antimicrobial resistance” and 84% (51/61) agreed that antimicrobial resistance is an important problem at UCI. Multiple factors were felt to contribute to antimicrobial resistance including the use of too many antibiotics, patient insistence on antibiotics, and poor patient adherence (Fig 1). While 72% (44/61) had heard of the term “antimicrobial stewardship”, only 25% (15/61) knew a lot about what it meant. Numerous factors were considered important to antibiotic decision-making including patient white blood cell count and severity of illness (Fig 2). Perceived barriers to infection diagnosis included the inability to obtain blood cultures and to regularly measure patient temperatures; perceived barriers to obtaining blood cultures included patient cost and availability of supplies (Fig 3).

Figure 1. Factors that doctors, pharmacists, and nurses working at the Uganda Cancer Institute (UCI) perceive as contributing to antimicrobial resistance at the UCI.

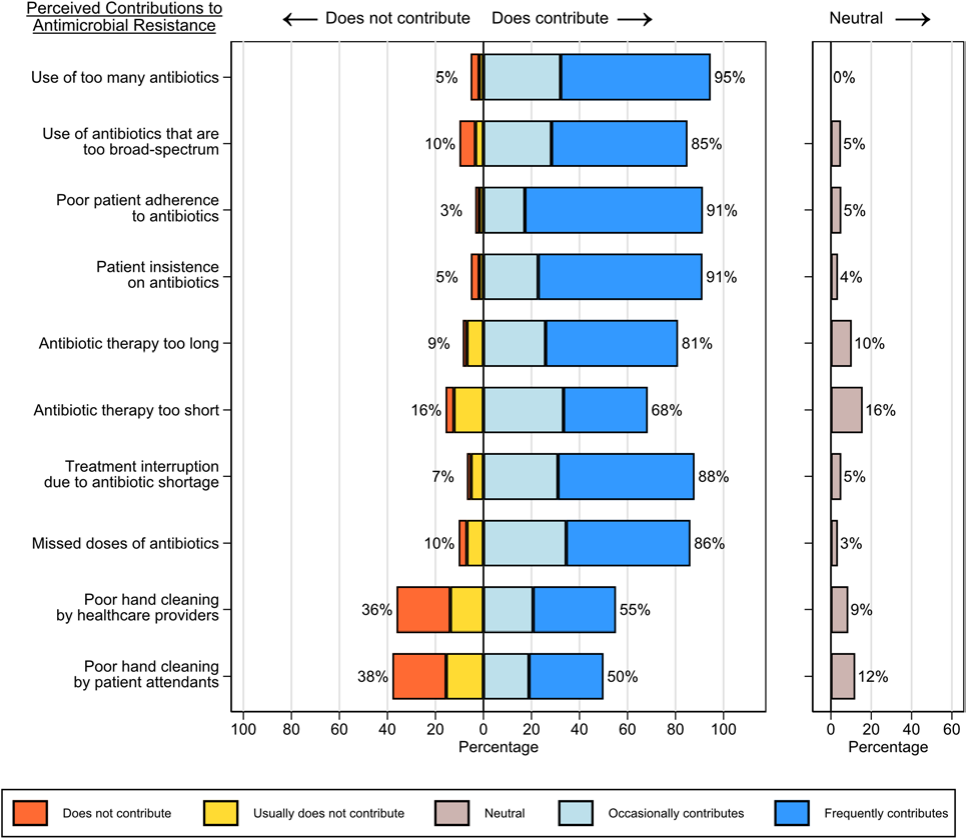

Percentages shown next to bars represent the combined total percentage of respondents reporting that the factor does not or usually does not contribute (left of bars, main chart), occasionally or frequently contributes (right of bars, main chart), or neither contributes nor does not contribute (right of neutral chart).

Figure 2. Factors that doctors, pharmacists, and nurses working at the Uganda Cancer Institute consider to be important when choosing antibiotics to treat infections.

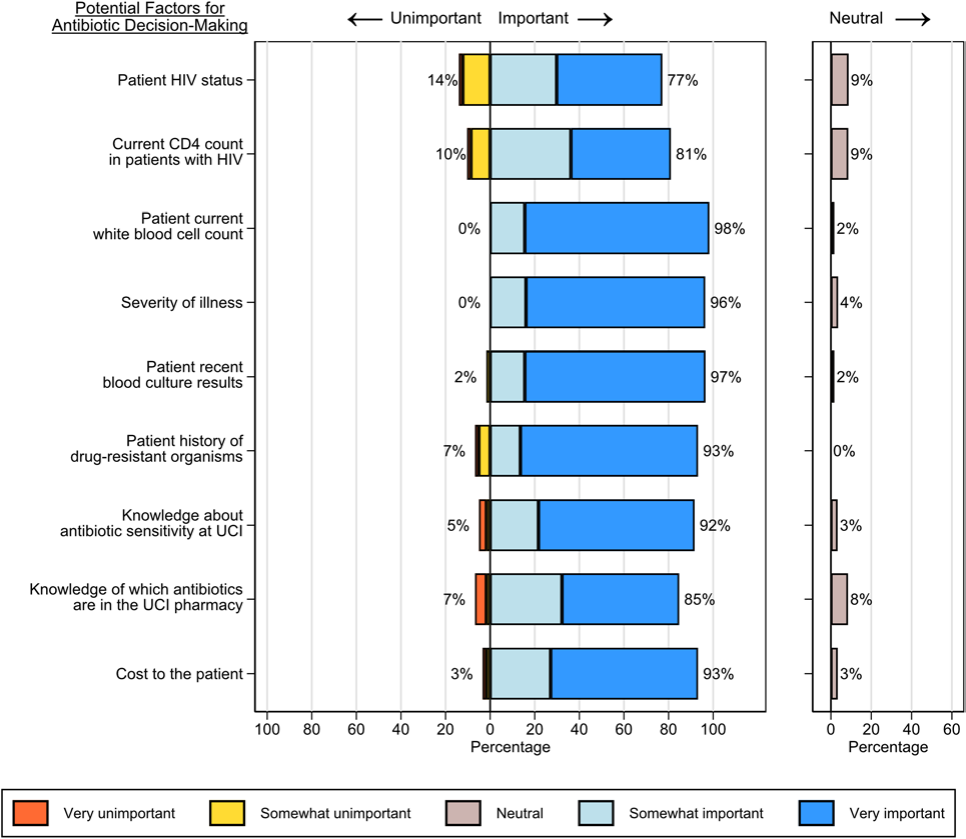

Percentages shown next to bars represent the combined total percentage of respondents reporting that the factor is somewhat or very unimportant (left of bars, main chart), somewhat or very important (right of bars, main chart), or neither important nor unimportant (right of neutral chart).

Figure 3. Factors that doctors, pharmacists, and nurses working at the Uganda Cancer Institute perceive as limiting the ability to diagnose infections and obtain blood cultures.

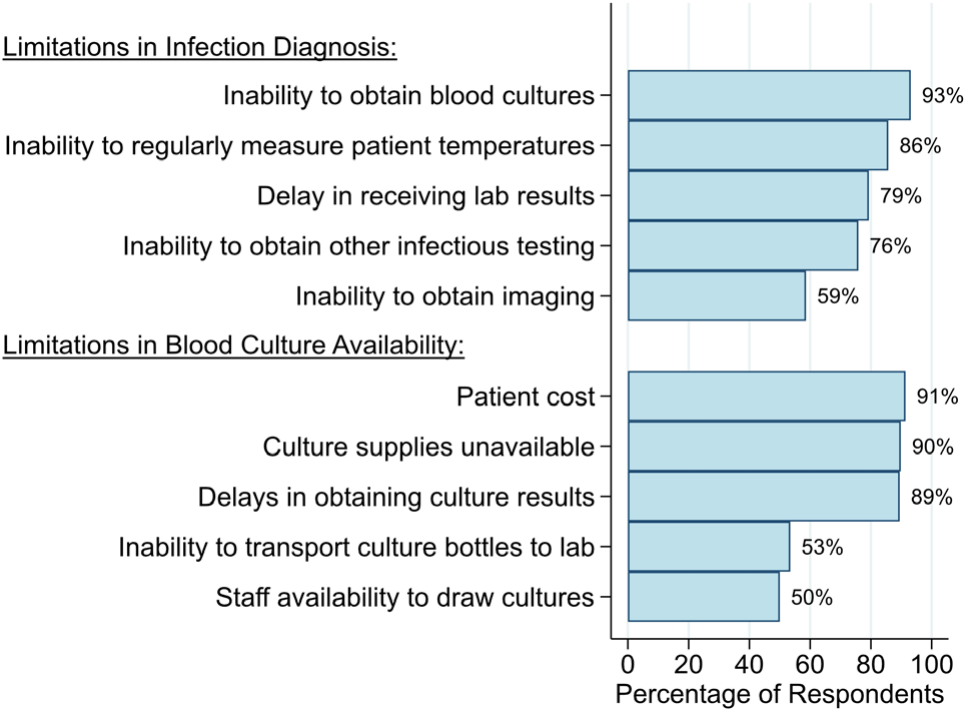

**Conclusion:**

While most staff recognized the term “antimicrobial resistance” and identified this as a major local problem, fewer were familiar with the term “antimicrobial stewardship”. We identified numerous perceived barriers to infection diagnosis and treatment, including the ability to consistently measure temperatures and the cost of blood cultures. A multipronged approach is needed to improve staff knowledge of antimicrobial stewardship and to address the systematic barriers to infection management at UCI.

**Disclosures:**

**All Authors**: No reported disclosures

